# Whole genome sequencing identifies genetic variants associated with senescence-accelerated phenotype of OXYS rats

**DOI:** 10.18699/vjgb-26-90

**Published:** 2026-10

**Authors:** V.M. Petrova, M.S. Protasova, A.D. Manakhov, N.A. Stefanova, N.G. Kolosova, E.I. Rogaev

**Affiliations:** Sirius University of Science and Technology, Sirius Federal Territory, Krasnodar region, Russia; Sirius University of Science and Technology, Sirius Federal Territory, Krasnodar region, Russia Vavilov Institute of General Genetics of the Russian Academy of Sciences, Moscow, Russia; Sirius University of Science and Technology, Sirius Federal Territory, Krasnodar region, Russia Vavilov Institute of General Genetics of the Russian Academy of Sciences, Moscow, Russia; Institute of Cytology and Genetics of the Siberian Branch of the Russian Academy of Sciences, Novosibirsk, Russia; Institute of Cytology and Genetics of the Siberian Branch of the Russian Academy of Sciences, Novosibirsk, Russia; UMass Chan Medical School, Department of Psychiatry, Shrewsbury, United States

**Keywords:** premature aging, age-associated diseases, OXYS rat strain, whole genome sequencing, genetic variants, copy number variations, преждевременное старение, возраст-ассоциированные заболевания, линия крыс OXYS, полногеномное секвенирование, генетические варианты, вариации числа копий геномных регионов

## Abstract

Age is the primary risk factor for age-related diseases, the early onset of which manifests as premature aging, whereas delayed development forms the basis for healthy aging and longevity. Adverse genetic variants contribute to premature aging and its diverse manifestations, including increased multimorbidity. Identifying the genetic underpinnings of premature aging and developing preventive approaches are essential to extending the human healthspan, which requires fundamental research using animal models. In this study, we performed whole-genome sequencing (coverage depth >25×) of OXYS rats – a unique model of premature aging characterized by the early onset of geriatric disorders, including cataracts, AMD-like retinopathy, Alzheimer’s disease-like pathology, osteoporosis, sarcopenia, and hypertrophic cardiomyopathy – using the parental Wistar/Icgn rat strain as a control. The screening for single-nucleotide variants (SNVs), short insertions/deletions (indels), and copy number variations (CNVs) and comparative genomic analysis revealed genetic variants unique to OXYS rats that are absent in other rat strains. Specifically, we identified a large deletion in the Fat1 enhancer, deletions in the promoter regions of Abcf1 and Gsta6, and missense variants in Cic, Akap6, Nqo2, Cerkl, Bfsp1, and Cmya5 that lead to amino acid substitutions in highly conserved protein regions. Bioinformatic analysis suggested that these variants may contribute to the key features of premature aging in OXYS rats. Notably, deletions in the Fat1 enhancer and Gsta6 promoter, alongside missense substitutions in Cic, Akap6, and Nqo2, may drive early neurodegeneration and cognitive deficits by disrupting neurogenesis, reducing neuronal survival, and impairing antioxidant defense. The deletion in the Abcf1 promoter and the missense variants in Cerkl may induce retinopathy features by disrupting retinal homeostasis and weakening the protection of photoreceptors and retinal pigment epithelium (RPE) cells against oxidative stress and apoptosis. Furthermore, the missense substitution in Bfsp1 likely contributes to early cataract development by reducing the stability of lens intermediate filaments. Finally, missense variants in Cmya5 and Akap6 may promote sarcopenia and cardiomyopathy via the destabilization of myofibrils in skeletal muscles and cardiomyocytes (Cmya5) and through impaired skeletal muscle regeneration and structural disorganization of cardiomyocytes (Akap6).

## Abstract (ru)

Возраст – основной фактор риска ассоциированных со старением заболеваний, раннее развитие которых лежит в основе преждевременного старения, а более позднее – обеспечивает долголетие. Неблагоприятные генетические варианты способствуют преждевременному старению и его множественным проявлениям – увеличению числа коморбидных состояний. Установление предпосылок преждевременного старения и разработка подходов к его профилактике – основной резерв увеличения здорового периода жизни людей, что обусловливает необходимость в фундаментальных исследованиях на моделях животных. Нами проведено полногеномное секвенирование (с глубиной покрытия >25×) крыс линии OXYS – модели преждевременного старения, которое сопровождается ранним развитием комплекса гериатрических заболеваний: катаракты, ретинопатии, аналогичной возрастной макулярной дегенерации (ВМД), признаков болезни Альцгеймера (БА), остеопороза, саркопении и гипертрофической кардиомиопатии (контроль – родительская линия крыс Wistar/ Icgn). Поиск однонуклеотидных вариантов, инсерций-делеций (инделов) и вариаций числа копий геномных регионов (CNV) и сравнительный геномный анализ выявили генетические варианты, уникальные для крыс OXYS и не встречающиеся среди других линий крыс. Обнаружены: крупная делеция в энхансере гена Fat1; делеции в промоторных регионах генов Abcf1 и Gsta6; а также миссенс-варианты в генах Cic, Akap6, Nqo2, Cerkl, Bfsp1 и Cmya5, приводящие к аминокислотным заменам в высококонсервативных регионах белков. Проведенный биоинформатический анализ показал, что данные варианты, вероятно, вносят вклад в развитие ключевых проявлений преждевременного старения у линии крыс OXYS. А именно: делеции в энхансере Fat1 и промоторе Gsta6, а также миссенс-замены в Cic, Akap6 и Nqo2 могут способствовать ранней нейродегенерации и когнитивному дефициту, нарушая нейрогенез, снижая выживаемость нейронов и их антиоксидантную защиту; делеция в промоторной области Abcf1 и миссенс-варианты в Cerkl могут приводить к возникновению признаков ВМД, нарушая гомеостаз сетчатки и ослабляя защиту фоторецепторов и клеток пигментного эпителия сетчатки (РПЭ) от окислительного стресса и апоптоза соответственно, а миссенс-замена в Bfsp1 – к раннему развитию катаракты через снижение стабильности промежуточных филаментов хрусталика. Миссенс-варианты в Cmya5 и Akap6 могут способствовать развитию саркопении и кардиомиопатии: замена в Cmya5 – за счет дестабилизации миофибрилл в кардиомиоцитах и скелетных мышцах, а замена в Akap6 – за счет нарушения регенерации скелетных мышц и структурной организации кардиомиоцитов.

## Introduction

Age-related diseases are a natural consequence of the aging process. Their onset at an earlier age is a hallmark of premature aging, whereas their delayed manifestation forms the basis of healthy aging and longevity. Advances in the treatment of age-related diseases have led to a steady increase in life expectancy over the last few decades, while the healthy lifespan is increasing at a noticeably slower rate. Currently, over 60 % of individuals aged 65 and older suffer from multimorbidity rather than a single geriatric disorder (Figueira et al., 2016), the variety and age of onset of which are determined by genetic and environmental factors, quality of life, and access to healthcare. In this context, identifying the molecular and genetic underpinnings of premature aging and developing reliable methods for its prevention are fundamental to extending the human healthspan.

Research in this field requires fundamental studies using biological models. One of them is the OXYS rat strain (Institute of Cytology and Genetics, Siberian Branch of the Russian Academy of Sciences, hereafter ICG SB RAS), a model of premature aging characterized by the early development of a complex of geriatric diseases (Kolosova et al., 2014). The strain was bred in the 1970s by selecting Wistar/Icgn rats for early spontaneous cataracts. In the first five generations, cataract development was induced by galactose loading; subsequently, selection was carried out for early spontaneous cataracts, with which the animals inherited the premature aging syndrome.

Against a background of moderately elevated blood pressure, OXYS rats spontaneously develop cataracts, hypertrophic cardiomyopathy, sarcopenia, osteoporosis, and a retinopathy similar to human age-related macular degeneration (Kolosova et al., 2014). OXYS rats also develop all the key pathogenetic and “clinical” features of Alzheimer’s disease (AD) in the absence of mutations in the genes (App, Psen1, and Psen2) specific to the familial form of AD. Thus, the strain can be considered a model of the most common (>95 %) sporadic form of this disease (Stefanova et al., 2014). The development of these manifestations of premature aging could be linked to oxidative stress, an imbalance in the systems responsible for the generation and detoxification of reactive oxygen species (ROS). However, elevated levels of oxidative damage to lipids, proteins, and DNA are recorded in tissues of OXYS rats later than signs of premature aging become apparent (Kolosova et al., 2006). Mitochondrial dysfunction, initially identified in the liver (Kolosova et al., 2001) and subsequently in muscles (Vays et al., 2014), the heart muscle (El’darov et al., 2015), brain structures (Tyumentsev et al., 2018), and the retina (Zhdankina et al., 2023), is considered the most likely cause of premature aging in OXYS rats. The link between mitochondrial dysfunction and premature aging in OXYS rats is also supported by the presence of single-nucleotide variants (SNVs) that can cause abnormalities in protein structure and/or function and influence the development of the OXYS phenotype (Devyatkin et al., 2020a, b).

The aim of this study is to identify genetic variants of various types – SNVs, short insertions/deletions (indels), and copy number variations (CNVs) – that may be associated with the development of the premature aging phenotype in OXYS rats.

## Materials and methods

Animals. The study was performed on male rats of the OXYS strain (n = 3) and the Wistar/Icgn parental strain (n = 3) provided by the Center for Genetic Resources of Laboratory Animals at ICG SB RAS. The animals were kept under standard laboratory conditions (22 ± 2 °C and a 12-hour light/dark cycle) in cages (57 × 36 × 20 cm). Animals had ad libitum access to water and standard pelleted chow (BioPro, Russia).

Animal housing (including appropriate facilities, qualified personnel, and necessary documentation) and all animal experiments were conducted in accordance with the ethical guidelines for the use of animals in research supported by the Russian Science Foundation. The study also complied with Directive 2010/63/EU of the European Parliament and Council of the European Union of September 22, 2010, and was approved by the Bioethics Committee of ICG SB RAS (Protocol No. 85/1 of June 18, 2021).

Tissue collection. Animals were euthanized using carbon dioxide asphyxiation followed by decapitation for tissue harvesting. Cerebellar tissue fragments (30 mg) were collected from 3-month-old OXYS (n = 3) and Wistar/Icgn (n = 3) rats into 1.5 mL tubes. The samples were immediately frozen in liquid nitrogen and stored at –80 °C.

DNA extraction. Genomic DNA was extracted using the QIAamp DNA Mini Kit (Qiagen, Germany). DNA quality and quantity were assessed using a NanoDrop OneC microvolume UV-Vis spectrophotometer (Thermo Fisher Scientific, USA) and a Qubit 4 fluorometer (Thermo Fisher Scientific, USA).

Library preparation and whole-genome sequencing. Whole-genome libraries were prepared using the TruSeq DNA PCR-Free Kit (Illumina, USA). Library quality was assessed using a TapeStation 4150 automated electrophoresis platform (Agilent Technologies, USA), and library molarity was determined by quantitative PCR using the KAPA Library Quantification Kit (Roche Sequencing Solutions, USA). Paired-end sequencing (2 × 151 bp) was performed on a NovaSeq 6000 system (Illumina, USA).

Sequencing data processing. The quality of the obtained reads was assessed using FastQC v0.11.9 (Andrews, 2010). Adapter removal and quality filtering were performed using AdapterRemoval v2.3.3 with the parameters --minquality 20, --trimqualities, and --minlength 50 (Schubert et al., 2016). After preprocessing, the reads were mapped to the mRatBN7.2 Rattus norvegicus genome assembly using the BWA-MEM algorithm from BWA v0.7.17 (Li H., Durbin, 2009). Read duplicates were marked using MarkDuplicates from Picard v2.26.11 (Broad Institute, 2022). Alignment quality was assessed using SAMtools v1.9 (Li H. et al., 2009). Sequencing coverage was estimated using the CollectWgs- Metrics tool from Picard v2.26.11 (Broad Institute, 2022).

Identif﻿ication, annotation and filtering of SNVs and indels. The positions and genotypes of SNVs and indels were identified using HaplotypeCaller from GATK v4.2.2.0 (McKenna et al., 2010). Principal component analysis (PCA) was performed using PLINK v1.9 (Purcell et al., 2007) and visualized in RStudio (R version 4.3.2) using the tidyverse and ggplot2 packages (Wickham, 2016; Wickham et al., 2019). Genotype filtering was performed using a custom Python script. The script accepted a VCF file as input to select variants with a minimum coverage of 10×, homozygous for the alternative allele in all samples of the case group (OXYS) and absent in the homozygous state from the control group (Wistar/Icgn).

The Ensembl Variant Effect Predictor (VEP) version 113 (McLaren et al., 2016) was used for the annotation and classification of each SNV and indel. The SNV and indel genotypes of OXYS rats were compared with those of 146 rat strains from the Rat Genome Database (RGD) (de la Cruz et al., 2004) using BCFtools v1.15 (Danecek et al., 2021). All selected genetic variants were manually inspected using the Integrative Genomics Viewer (IGV) (Robinson et al., 2011). Analysis of amino acid substitutions was performed for canonical gene transcripts according to the Ensembl version 115 annotation (Dyer et al., 2025). The conservation of amino acid residues was assessed based on multiple alignments of orthologs from placental mammals (Table S1 in Supplementary Materials)1, rodents, and related species (Table S2), retrieved from the Ensembl database version 115 (Dyer et al., 2025). A search for paralogs was also conducted in the Ensembl database version 115 (Dyer et al., 2025). Multiple alignments were visualized using UGENE v45.0 software (Okonechnikov et al., 2012). Protein structure prediction and visualization were performed in AlphaFold 3 (Abramson et al., 2024).

Supplementary Materials are available in the online version of the paper: https://vavilov.elpub.ru/jour/manager/files/Suppl_Petrova_Engl_30_6.pdf


CNV detection, annotation and selection. CNV detection was performed using CNVpytor v1.3.1 with a 100-bp bin size as recommended for 20–30× coverage (Abyzov et al., 2011; Suvakov et al., 2021). Smoove v0.2.8 (Pedersen et al., 2020) was used to refine CNV coordinates and genotype the samples. For subsequent annotation, CNVs in regions with low mapping quality were filtered out. Genotypes were filtered using BCFtools v1.15 (Danecek et al., 2021). CNV annotation was performed by intersecting the coordinates with the genomic features of the mRatBN7.2 Rattus norvegicus assembly using the intersect command from BEDTools v2.30.0 (Quinlan, Hall, 2010). BEDTools v2.30.0 was also used to intersect CNV coordinates with H3K27ac enrichment peaks generated by MACS2 v2.2.6 (Zhang et al., 2008). CNV visualization was performed using IGV (Robinson et al., 2011).

A schematic description of the bioinformatics analysis is presented in Figure 1.

**Fig. 1.**
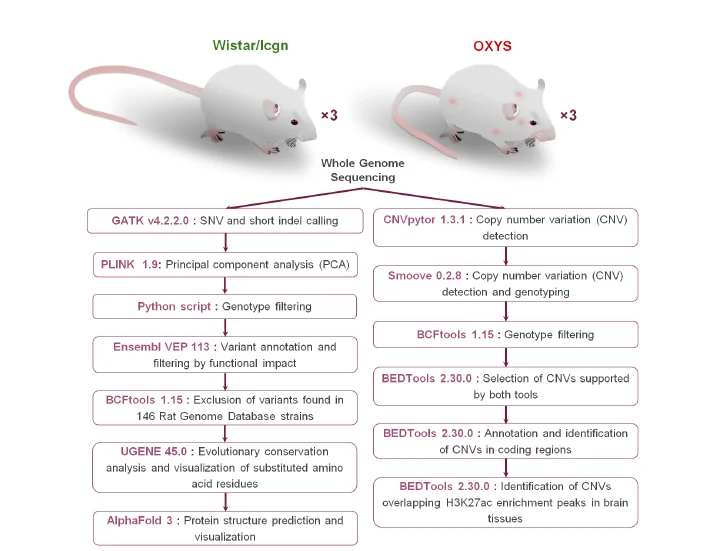
Bioinformatics pipeline for genome analysis of Wistar/Icgn and OXYS rats.

## Results

**Principal component analysis (PCA)**

The genome coverage depth for each OXYS and Wistar/Icgn rat sample is presented in Table S3. The mean coverage depth was 32.36× for OXYS and 28.88× for Wistar/Icgn rats. A total of 12,412,404 genetic variants differing from the mRatBN7.2 Rattus norvegicus reference assembly were identified in the genomes of OXYS and Wistar/Icgn rats.

The genotypes of the OXYS and Wistar/Icgn rats were subjected to PCA. The two-dimensional plot shows the first (PC1) and second (PC2) principal components, which together account for 79.1 % of the total variance (Fig. 2). The plot demonstrates that the OXYS and Wistar/Icgn samples, as expected, form distinct clusters. Notably, the OXYS rat cluster (marked in red) is tightly grouped, pointing to a genetic homogeneity characteristic of inbred strains.

**Fig. 2.**
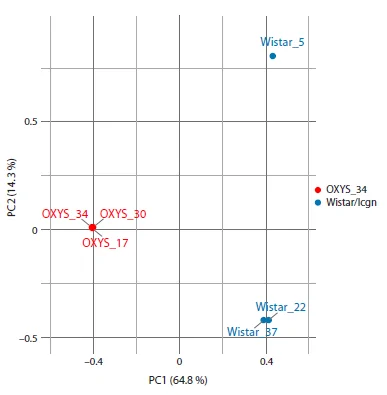
Principal component analysis (PCA) performed using PLINK v1.9 based on the genotypes of OXYS (red circles) and Wistar/Icgn (blue circles) rats.

**Analysis of SNVs and indels**

Since the OXYS rat strain is inbred, the genetic variants determining its specific phenotype were expected to be homozygous in all individuals. Out of 12,009,286 chromosomal variants, we selected 1,363,823 positions where all three OXYS rats were homozygous for the alternative allele (with a coverage depth of ≥10×), while no individuals from the control Wistar/ Icgn strain carried this allele in a homozygous state. Variants leading to predicted high-impact functional disruptions in canonical gene transcripts were then selected according to the Sequence Ontology classification (Eilbeck et al., 2005). Specifically, 78 variants were identified as causing premature translation termination, loss of gene function, or triggering nonsense-mediated mRNA decay. Additionally, 423 missense variants predicted as damaging by the SIFT algorithm and leading to amino acid substitutions in the translated proteins were selected. By comparing the SNV and indel genotypes of OXYS rats with the genotypes of 146 rat strains (List S1) from RGD (de la Cruz, 2004), we identified 33 high-impact variants and 87 damaging missense variants that were completely absent from all other strains.

From this pool of genetic variants unique to OXYS rats, we prioritized variants in genes involved in key pathways related to aging and age-related pathologies, such as mitochondrial dysfunction, oxidative stress, neurodegeneration, cognitive and visual impairment, sarcopenia, and lipid metabolism disorders. Each candidate variant was verified by visual inspection of alignments using IGV and by analyzing the evolutionary conservation of the affected amino acid residues. Variants in regions with poor mapping quality, alignment artifacts, or low conservation were rejected. As a result, the following candidate genes were selected: Abca1, Apob, Cic, Nqo2, Akap6, Cmya5, Cerkl, and Bfsp1, whose functional impairments may underlie the premature aging phenotype of OXYS rats. The variants identified in these candidate genes are summarized in Table 1, visualized using IGV (Fig. S1), and shown in the transcript models (Fig. S2).

**Table 1.**
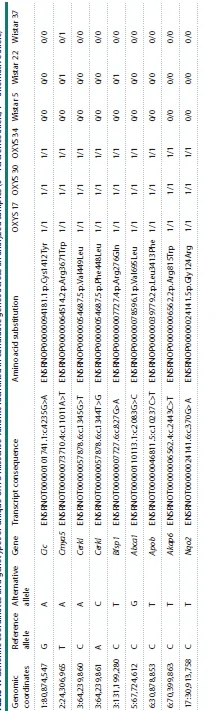
Genomic coordinates and genotypes of unique OXYS missense variants identified in candidate genes across all analyzed samples (0 – reference allele, 1 – alternative allele)

**Lipid metabolism disorders.** The Apob and Abca1 genes are involved in lipid metabolism. Abca1 is predominantly expressed in rat adipose tissue and liver, as well as in various immune cell types (Wu et al., 2009; RatGTEx Consortium, 2021). The Abca1 gene product plays a key role in transporting cholesterol and phospholipids across the cell membrane, which is essential for high-density lipoprotein (HDL) formation in the liver (Oram, 2003; Ghanbari-Niaki et al., 2007). The V695L amino acid substitution is located in one of the transmembrane domains of Abca1, which is enriched in hydrophobic amino acid residues (Fig. 3A and Fig. S3). Valine (V) is present at the homologous position in most of its orthologs among placental mammals and rodents; however, leucine (L) was identified in the Arctic ground squirrel (Urocitellus parryii) (Fig. S4A). Other hydrophobic amino acids can also be found at this position. Examples are methionine (M) in birds, including the chicken (Gallus gallus) and the golden eagle (Aquila chrysaetos), and isoleucine (I) in fish, such as the Atlantic salmon (Salmo salar) (Fig. 3A). Furthermore, Abca1 has 15 ancient paralogs, which, in addition to valine (V), contain other hydrophobic amino acids at the homologous position, including isoleucine (I), alanine (A), methionine (M), and leucine (L) (Fig. S5). Taken together, these data indicate that the V695L substitution is likely compatible with the structural integrity and function of the protein.

**Fig. 3.**
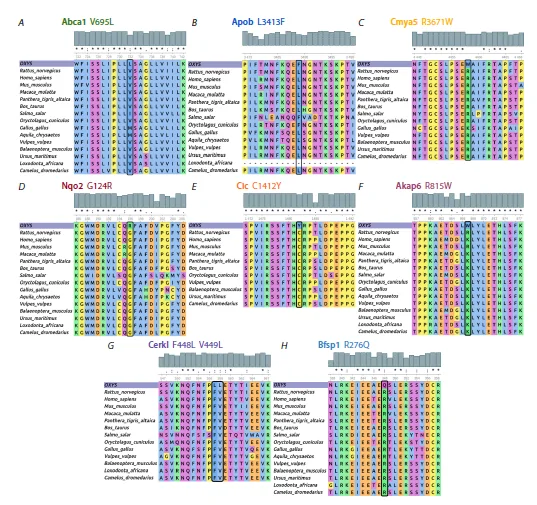
Amino acid substitutions in OXYS rat proteins (top of each panel) and the homologous position (indicated by a black box) across orthologous proteins from various animal species, including the brown rat (Rattus_norvegicus), human (Homo_sapiens), house mouse (Mus_musculus), rhesus macaque (Macaca_mulatta), Amur tiger (Panthera_tigris_altaica), cattle (Bos_taurus), Atlantic salmon (Salmo_ salar), European rabbit (Oryctolagus_cuniculus), chicken (Gallus_gallus), golden eagle (Aquila_chrysaetos), red fox (Vulpes_vulpes), blue whale (Balaenoptera_musculus), polar bear (Ursus_maritimus), African bush elephant (Loxodonta_africana), and dromedary camel (Camelus_dromedarius). Amino acids are colored by chemical properties: polar uncharged (S, T, N, Q) in purple; hydrophobic (A, V, I, L, M, F, Y, W) in blue; positively charged (R, H, K) in green; negatively charged (D, E) in orange; and special cases (C, G, P) in yellow. The evolutionary conservation track is located above the sequences as a bar chart representing the degree of conservation. Images were generated using UGENE v45.0 software (Okonechnikov et al., 2012).

The Apob gene encodes two isoforms of apolipoprotein B: ApoB-100, a structural protein of low-density lipoprotein (LDL) synthesized in the liver, and ApoB-48, a component of chylomicrons produced by small intestinal cells. ApoB-100 is involved in the synthesis and secretion of very-low-density lipoprotein (VLDL), the precursor of LDL required for the transport of endogenous triglycerides and cholesterol into the bloodstream (Davidson, Shelness, 2000). Furthermore, ApoB-100 serves as a ligand for LDL receptors on hepatocytes, mediating the uptake and clearance of ApoB-containing particles by the liver (Davidson, Shelness, 2000). The Apob gene has no paralogs. The identified amino acid substitution L3413F is located within the heparin-binding domain of ApoB-100, which mediates the binding of LDL particles to glycosaminoglycan-rich proteoglycans of the extracellular matrix (Flood et al., 2004) (Fig. 3B and Fig. S6). While leucine (L) is present at the homologous position in most rodents and mammals, phenylalanine (F) was found at this position in the European rabbit (Oryctolagus cuniculus) and the Atlantic salmon (S. salar) (Fig. 3B and Fig. S4B). Since both amino acids are hydrophobic and phenylalanine occurs at the homologous position in evolutionarily distant species without causing a loss of viability, it can be concluded that the L3413F substitution does not disrupt protein function significantly. However, because phenylalanine possesses a bulky aromatic side chain, its potential impact on the local conformation of the domain or the efficiency of interaction with extracellular matrix proteoglycans cannot be entirely ruled out. Therefore, this substitution likely represents a variant with low functional impact.

**Skeletal and cardiac muscle dysfunctions.** The Cmya5 gene encodes a large protein highly expressed in skeletal muscles and the heart, where it is involved in structural organization of sarcomeres in cardiomyocytes and skeletal muscle fibers (Wu et al., 2009; Lu et al., 2022). The identified missense variant results in an R3671W substitution within the Fibronectin type-III 1 domain (FN3-1) of Cmya5, which mediates interactions with titin M-domains and stabilizes the sarcomere (Sarparanta, 2008; Sarparanta et al., 2010) (Fig. 3C and Fig. 4A). Arginine (R) at this position is highly conserved across evolutionarily distant animal species, as well as among rodents (Fig. 3C and Fig. S4C). Lysine (K), which carries a positive charge similar to arginine, is found only in the chicken (G. gallus). The Cmya5 protein has three evolutionarily ancient paralogs: Fsd1, Fsd2, and Fsd1l. One of these, Fsd2, is expressed in muscle tissue, but it performs different functions. The substitution of positively charged arginine for hydrophobic aromatic tryptophan at position 3671 (R3671W) is located within a β-sheet of the Cmya5 FN3-1 domain. This mutation disrupts local interactions with neighboring amino acid residues, presumably impairing domain folding and, as a consequence, its interactions with other proteins (Fig. 4A).

**Neurodegeneration and cognitive decline.** The Nqo2 gene encodes the flavin-dependent protein Nqo2, which is structurally homologous to Nqo1 quinone dehydrogenase but differs significantly in cofactor specificity. Nqo2 binds the physiological cofactor NAD(P)H extremely inefficiently and likely functions primarily as a regulatory rather than a catalytic protein (Islam, Shilton, 2025). Nqo2 is highly expressed in the kidney and liver (Wu et al., 2009; RatGTEx Consortium, 2021). The missense variant detected in OXYS rats results in a G124R substitution at a position highly conserved across evolutionarily distant animals, rodents, and related species (Fig. 3D and Fig. S4D). This substitution is located near the FDV (phenylalanine-aspartic acid-valine) substrate-binding site at positions 127–129 (Fig. 4B). Nqo2 has two paralogs: a predicted gene, AABR07044714, which arose in rats, and the evolutionarily more ancient Nqo1, which encodes an enzyme responsible for quinone reduction. The substitution of a small glycine for a bulky, positively charged arginine disrupts local interactions within the protein and may affect protein–protein interactions, ligand affinity, and the redox-dependent regulatory function of Nqo2 (Fig. 4B).

**Fig. 4.**
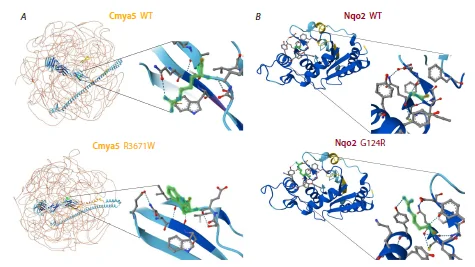
Protein structures predicted using AlphaFold 3 (Abramson et al., 2024). A – Cmya5 (wild-type, WT (3671R); and with the OXYS rat substitution, R3671W). The inset shows a magnified view of the FN3-1 domain harboring the substitution. The amino acid residue at position 3671 is highlighted in green; B – Nqo2 (wild-type, WT (124G); and with the OXYS rat substitution, G124R). The inset shows a magnified view of the substitution site. The amino acid residue at position 124 is highlighted in green. The color coding reflects the local confidence of the structure prediction (pLDDT): blue (90–100) indicates very high, light blue (70–89) indicates confident, yellow (50–69) indicates low, and orange (< 50) indicates very low. Regions with pLDDT < 50 likely represent intrinsically disordered protein regions (IDRs).

The Cic gene encodes the Capicua transcriptional repressor, with its highest expression observed in nervous tissue (RatGTEx Consortium, 2021). It plays a crucial role in neurogenesis and acts as a suppressor for various tumor types (Wong, Yip, 2020; Hourigan et al., 2021). The C1412Y substitution identified in OXYS rats is located within a highly conserved region of the protein, which, however, lacks an ordered domain structure and is predicted with low confidence (pLDDT < 50) (Fig. 3E and Fig. S7). All analyzed orthologs possess a cysteine (C) residue at the homologous position (Fig. 3E and Fig. S4E). Cic has only one ancient paralog, Bbx, which performs distinct functions and lacks the homologous region. Since the C1412Y substitution occurs within a highly conserved region of the protein and its functional compensation by the Bbx paralog is impossible, this variant may impair the functional activity of the Cic protein despite its location within a structurally disordered region.

The Akap6 gene encodes an adapter protein that anchors signaling complexes to the nuclear envelope and sarcoplasmic reticulum (Dodge-Kafka et al., 2005; Vergarajauregui et al., 2020). In rats, it is expressed primarily in the heart, skeletal muscle, and brain, including the hippocampus and cerebral cortex (Wu et al., 2009; RatGTEx Consortium, 2021). The identified R815W substitution is located within the Spectrin 1 repeat domain (SR1) of Akap6, which is critical for protein– protein interactions (Vergarajauregui et al., 2020) (Fig. 3F and Fig. S8). The Akap6 protein has one paralog, Cep68, which lacks the homologous region. It does not perform similar adapter functions. Analysis of multiple ortholog alignments demonstrates that position 815 is strictly conserved in charge. Either arginine (R) or lysine (K) – both positively charged amino acid residues – is present at the homologous position across all analyzed animal species (Fig. 3F and Fig. S4F). Thus, the R815W substitution is nonconservative: a positively charged polar arginine is replaced by a bulky hydrophobic tryptophan residue containing an aromatic ring, which may disrupt protein–protein interactions mediated via the SR1 domain.

Visual impairments. The Cerkl gene is involved in regulating autophagy, mitochondrial dynamics, and metabolism. It plays a key role in protecting the photoreceptors, and retinal pigment epithelial (RPE) cells against oxidative stress and apoptosis (Mirra et al., 2021). Two consecutive amino acid substitutions, F448L and V449L, identified in Cerkl are located within the disordered C-terminal region of the protein, which is enriched in hydrophobic amino acid residues (Fig. 3G and Fig. S9). Phenylalanine (F) at position 448 is strictly conserved among all analyzed orthologs (Fig. 3G and Fig. S4G). However, valine (V) at position 449 tolerates a replacement with another hydrophobic amino acid, isoleucine (I), in rodents (Fig. S4G). The Cerkl protein has four evolutionarily ancient paralogs (Cerk, Sphk1, Sphk2, and Agk); however, unlike Cerkl, they function as active lipid kinases and utilize different regulatory mechanisms. Nevertheless, because both substitutions are located within a region enriched in hydrophobic residues, their impact on the structure and function of the Cerkl protein may be minimal.

In the Bfsp1 gene, which encodes a structural protein of lens intermediate filaments, the missense substitution leading to R276Q was identified within one of the α-helical domains of the rod region (Fig. 3H and Fig. S10). This region ensures filament polymerization and interaction with the Bfsp2 protein to form the cytoskeleton of lens fibers (Perng et al., 2007). Arginine (R) at position 276 is highly conserved among the analyzed orthologs (Fig. 3H and Fig. S4H). However, in the Chinese hamster (Cricetulus griseus), tryptophan (W) is present at the homologous position (Fig. S4H). The Bfsp1 gene has no paralogs, which points that its function is unique. Despite the overall preservation of the protein structure, the loss of positive charge due to the glutamine-for-arginine-substitution in the center of one of the rod region domains may disrupt intermediate filament assembly in the lens.

Visualization of the tissue-specific expression for each of the aforementioned genes in rats is presented in Figures S11–S18.

Mitochondrial dysfunction. The premature aging phenotype of OXYS rats is considered to be associated with mitochondrial dysfunction (Tyumentsev et al., 2018). Consequently, variants in genes associated with the mitochondrial function were selected from the pool of missense substitutions and high-impact functional variants distinguishing OXYS rats from Wistar/Icgn. Missense substitutions predicted to be damaging by the SIFT algorithm were detected in the Mrps30, Decr1, Mecr, Ptcd1, and Afg3l1 genes (Table S4). The analysis of the prevalence of these genetic variants across 146 rat strains (List S1) showed that they occurred with high frequency among other strains, including healthy ones (Table S5). Therefore, they likely do not contribute significantly to the development of structural and functional disorders in the mitochondria of OXYS rats.

**Copy number variation (CNV) analysis**

To improve the accuracy of CNV detection and breakpoint identification, two software tools employing different approaches were utilized: CNVpytor (based on a read depth algorithm) and Smoove (based on split-read and discordant paired-end read algorithms). The majority of CNVs present in the OXYS rat genomes and absent from the control Wistar/ Icgn strain were located within noncoding regions, specifically intergenic regions and introns. Following the selection of functionally significant CNVs (overlapping coding or regulatory regions), only deletions remained. No other structural variation types were detected within functionally significant regions.

**CNVs in coding regions.** Some of the CNVs identified in the OXYS rat genomes affected coding regions, specifically the exons of the LOC100911093, Ybx1l1, Gsta6, and Mettl24 genes (Table 2). The LOC100911093 and Ybx1l1 genes were found to be completely deleted (Fig. 5). LOC100911093 presumably encodes mitochondrial CCA-tRNA nucleotidyltransferase and has one paralog in rats, Trnt1, which shares 99.54 % sequence identity and performs the same function within both the cytoplasm and mitochondria. Ybx1l1 is predicted to bind nucleic acids and participate in the regulation of gene expression. Ybx1l1 has six paralogs, including Ybx1, which exhibits high sequence identity (94.48 %) to Ybx1l1. Its human ortholog, YBX1, is known to be involved in DNA repair under oxidative stress (Zheng et al., 2025). Both LOC100911093 and Ybx1l1 remain poorly characterized in rats, which hampers the assessment of the functional consequences of their loss. However, the presence of highly identical paralogs (Trnt1 and Ybx1) suggests a potential for partial functional compensation.

**Table 2.**
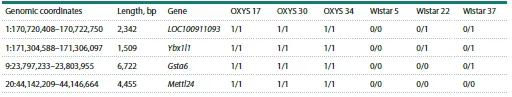
Genomic coordinates and genotypes of deletions affecting the coding regions (exons) of genes across all analyzed samples (0 – reference allele, 1 – deletion)

**Fig. 5.**
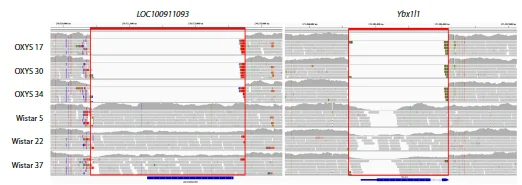
Deleted LOC100911093 and Ybx1l1 genes in the OXYS rat genomes. Scale bar: 3,500 bp. Red boxes indicate the deletion boundaries. Images were generated using IGV (Robinson et al., 2011).

The first exons of both the Gsta6 and Mettl24 genes fall within the deletion regions. The enzyme encoded by the Gsta6 gene belongs to the alpha glutathione S-transferase family and participates in the metabolism of glutathione, an antioxidant that plays a key role in neutralizing free radicals and maintaining cellular redox homeostasis (Forman et al., 2009). This gene remains poorly characterized and has 19 paralogs, five of which also belong to the alpha glutathione S-transferase family, potentially providing partial functional compensation for a possible dysregulation of Gsta6. Mettl24 encodes an enzyme with predicted methyltransferase activity but has been poorly characterized in rats. Its human ortholog, METTL24, is implicated in immune functions and associated with kidney renal clear cell carcinoma (KIRC) (Jiang et al., 2022). The Mettl24 gene has two ancient paralogs, Scara3 and Cthrc1, which share low sequence identity with the query gene (7.76 and 16.97 %, respectively) and are not functionally related to methyltransferases.

CNVs in regulatory genomic regions. H3K27ac is an epigenetic modification (acetylation) of lysine 27 in the N-terminal region of the DNA packaging protein histone H3. The enrichment of a genomic region in this epigenetic mark indicates the location of active regulatory elements, such as promoters and enhancers. It has been shown that the development of AD traits in OXYS rats, as in humans, is associated with neurodegenerative changes primarily in the hippocampus and prefrontal cortex (Stefanova et al., 2014). Therefore, we investigated whether the CNVs identified in the OXYS rat genomes fall within regions of active regulatory elements in the ventral hippocampus, dorsal hippocampus, and prefrontal cortex.

By using ChIP-seq data from the respective brain regions of control Long-Evans outbred rats (Rayan et al., 2022), the coordinates of enriched peaks for the H3K27ac histone mark were obtained and found to intersect with the coordinates of the unique OXYS CNVs. Deletions within the Gsta6, Mettl24, Fat1, and Babam2 genes, as well as upstream of the Abcf1 gene, overlap with H3K27ac-enriched regions (Table 3 and Fig. 6). The above-described deletions within the Gsta6 and Mettl24 genes are located in their putative promoter regions, and they affect the first exon. The deletions within Fat1 and Babam2 are located within introns and are likely to disrupt their active enhancers. The Fat1 gene encodes a protein from the atypical cadherin family, which is involved in cell–cell adhesion, muscle and neuronal development, and the positive regulation of the migration of various cell types, including neurons, fibroblasts, astrocytic precursors, and vascular smooth muscle cells (Riascos-Bernal et al., 2023; Helmbacher, 2018). Fat1 has seven paralogs, three of which also belong to the atypical cadherin family. The Babam2 gene encodes an adapter protein that is a component of the BRCA1-A and BRISC complexes. It plays an important role in double-stranded DNA break repair, cell cycle maintenance, and the prevention of cellular senescence (Chung et al., 2020), while also inhibiting the mitochondrial apoptotic pathway (Li Q. et al., 2004). The absence of paralogs for the Babam2 gene precludes genetic compensation for potential dysregulation caused by the deletion within the enhancer region. Furthermore, the deletion upstream of the Abcf1 gene may affect its active promoter. The Abcf1 gene product is a member of the ATP-binding cassette (ABC) protein superfamily. It participates in the initiation and elongation of translation process and regulates the innate immune response (Wilcox et al., 2017). Both Abcf1 paralogs belong to the same ABC protein subfamily F.

**Table 3.**
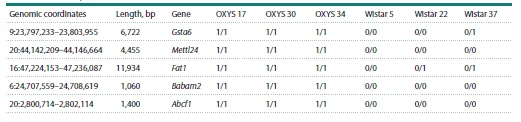
Genomic coordinates and genotypes of deletions overlapping H3K27ac enrichment peaks within the prefrontal cortex, dorsal hippocampus, and ventral hippocampus of Rattus norvegicus across all analyzed samples (0 – reference allele, 1 – deletion)

**Fig. 6.**
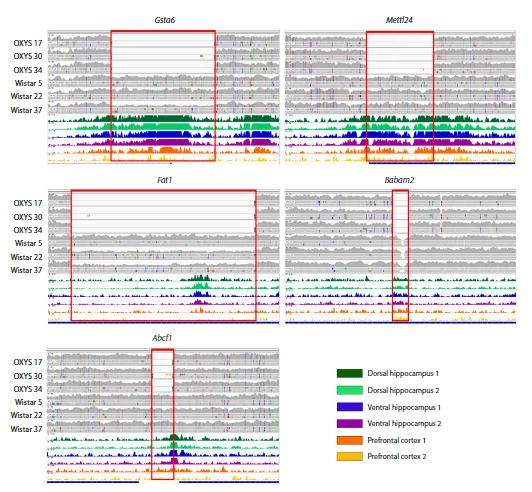
Deletions within the Gsta6, Mettl24, Fat1, Babam2, and Abcf1 genes overlapping H3K27ac enrichment peaks within the prefrontal cortex, dorsal hippocampus, and ventral hippocampus of Rattus norvegicus. Scale bar: 15 kb. Red boxes indicate the deletion boundaries. Images were generated using IGV (Robinson et al., 2011).

Our analysis indicates that the deletions identified in OXYS rats lead to the loss of gene regulatory regions. The Gsta6 and Mettl24 genes may be the most significantly affected, with deletions located in the most heavily H3K27ac-enriched regions that correspond to the active promoters of these genes.

Visualization of the tissue-specific expression for each of the aforementioned genes in rats is presented in Figures S19–S25.

## Discussion

The aim of this study was to identify genetic variants in OXYS rats that might contribute to the premature aging phenotype, which is accompanied by the development of various ageassociated pathologies.

**Cognitive impairment and neurodegeneration**

Variants detected within the Fat1, Cic, Akap6, Gsta6, and Nqo2 genes may be associated with the early development of neurodegenerative changes and cognitive decline in OXYS rats. A large deletion was identified in the noncoding region of the Fat1 gene, likely disrupting its active enhancer. In vertebrates, including rats, Fat1 is expressed in both the developing and adult brain, with high expression observed in regions involved in neurogenesis (Peng et al., 2021). Its product, FAT1, is involved in early stages of neuronal differentiation. It controls axon growth via the Hippo signaling pathway and promotes terminal cell differentiation by inhibiting cell proliferation (Ahmed et al., 2015; Peng et al., 2021). Thus, the identified deletion may reduce Fat1 expression in hippocampal neurogenesis zones, thereby disrupting the regulation of synaptic plasticity and neuronal migration, which could exacerbate the cognitive decline observed in OXYS rats.

The CIC (Capicua) protein is a key effector of the RTK/ RAS/MAPK signaling pathway, regulating the balance of proliferation and differentiation of neuronal progenitors in the hippocampus (Hourigan et al., 2021). It also acts as a tumor suppressor by repressing the transcription of ETS-family oncogenes (Wong, Yip, 2020) and forms a complex with the Ataxin-1 protein, which is associated with the development of the neurodegenerative pathology spinocerebellar ataxia type 1 (SCA1) (Rousseaux et al., 2018). Pathogenic variants within CIC have been described in human oligodendrogliomas, where its inactivation stimulates glial cell proliferation through the activation of ETV1/4/5 oncogenes (Yip et al., 2012). Impairment of the repressor function of CIC in the mouse hippocampus leads to a reduction in the pool of neuronal progenitors, alongside the disruption of their differentiation, migration, and dendritic development (Hourigan et al., 2021). Therefore, it can be hypothesized that the Cic missense variant found in OXYS rats contributes to the delay in brain maturation observed in this strain during the early postnatal period, which is associated with an altered balance of neural precursor proliferation and differentiation (Rudnitskaya et al., 2020).

The R815W substitution within the SR1 domain of Akap6 was previously described in a study of SNV transcriptome data from the hippocampus and prefrontal cortex of OXYS rats as associated with cognitive impairment (Devyatkin et al., 2020b). The spectrin repeats of AKAP6 are involved in binding to nuclear envelope proteins (nesprin-1α) and components of the microtubule-organizing center (pericentrin, AKAP9), and can also mediate the anchoring of signaling molecules such as calcineurin and NFATc4 (Mackiewicz et al., 2024). Disruption of the structural integrity of AKAP6 spectrin repeats may destabilize its anchoring to the nuclear envelope and the organization of perinuclear signaling complexes. This, in turn, may weaken pro-survival signaling from BDNF receptors to transcriptional targets (e. g., via the calcineurin-NFATc4 pathway), thereby increasing the vulnerability of neurons to stress and degeneration (Mackiewicz et al., 2024). Recent studies have demonstrated that AKAP6 plays a key role in neurotrophin-mediated survival of retinal ganglion cells (Tomczak et al., 2024). Furthermore, genome-wide association studies have linked polymorphisms within the AKAP6 locus to impairments in episodic memory, perceptual speed, and other cognitive functions in older adults (Andrews et al., 2017). The location of R815W within the functionally significant SR1 domain and the evolutionary conservation of positive charge at the homologous position in AKAP6 orthologs suggest that this variant may contribute to the development of cognitive decline in OXYS rats

A decrease in Gsta6 (a member of the alpha glutathione S-transferase family) expression compared to control Wistar/ Icgn rats – previously detected in the prefrontal cortex, hippocampus, and retina of OXYS rats during postnatal brain development (Telegina et al., 2015; Stefanova, Kolosova, 2023) – may be caused by the deletion identified within the putative active promoter of this gene. The reduced Gsta6 expression may impair glutathione-dependent free radical scavenging, thereby weakening the antioxidant defense of neurons and photoreceptors.

Similarly, a missense substitution within the Nqo2 gene was previously described in a study of SNV transcriptome data from the hippocampus and prefrontal cortex of OXYS rats and characterized as potentially associated with neurodegeneration and AD development (Devyatkin et al., 2020b). NQO2 expression in the brain is recorded in areas critical for cognitive function, including the hippocampus and cortex, and it increases with age and AD progression (Janda et al., 2024). The role of NQO2 in learning and memory consolidation may be linked to its involvement in cellular metabolism and ROS production, including mitochondrial ROS (Islam, Shilton, 2025). Recent data also point to the involvement of NQO2 in the regulation of oxidative stress, inflammation, autophagy, and apoptosis, as well as to its ability to interact with a number of bioactive compounds, including certain anticancer drugs (Janda et al., 2024; Islam, Shilton, 2025). Thus, the identified genetic variants within Gsta6 and Nqo2 may contribute to the development of early neurodegenerative changes in the brain structures and retina of OXYS rats, which represent hallmarks of AD and AMD-like retinopathy.

It has been demonstrated that the frontal cortex and hippocampus of OXYS rats exhibit elevated levels of oxidative DNA damage (8-oxoguanine) compared to the parental Wistar/Icgn strain (Sattarova et al., 2013). The deletion identified within the Babam2 regulatory region is of interest as a potential factor contributing to genomic instability. Although there is no direct evidence of DNA double-strand break accumulation in OXYS rats, the reduced expression of repair genes may exacerbate the consequences of oxidative damage in the brain. Importantly, the antiapoptotic function of BABAM2 is mediated through the inhibition of the mitochondrial apoptotic cascade (Li Q. et al., 2004). Given that mitochondrial dysfunction and the dysregulation of apoptosis are early hallmarks of the OXYS phenotype (Stefanova et al., 2014), a reduced expression of Babam2 in the hippocampus and prefrontal cortex – driven by the detected deletion within the enhancer region – may increase the sensitivity of neurons to stress-induced death

**Ophthalmological pathologies: retinopathy and cataracts**

Clinical manifestations of retinopathy, resembling the dry form of age-related macular degeneration (AMD) in humans, appear in OXYS rats at the age of 3–4 months. They are accompanied by structural and functional changes in RPE cells and impaired choroidal microcirculation (Markovets et al., 2010). Pathological changes within the retina progress against the background of increased accumulation of beta-amyloid and lipofuscin in RPE cells, altered morphology, and reactive gliosis, ultimately leading to the complete loss of photoreceptors by 24 months of age (Markovets et al., 2010; Zhdankina et al., 2023). Several genetic variants identified in this study potentially contribute to the development of this pathology. These include a deletion within the putative promoter region of the Abcf1 gene, which plays a crucial role in retinal homeostasis by regulating the phagocytosis of photoreceptor outer segments by RPE cells (Guo et al., 2015). The detected deletion may reduce Abcf1 expression, thereby contributing to the development of AMDlike features in OXYS rats.

The Cerkl gene is a key protector of photoreceptors and RPE cells against oxidative stress and apoptosis (Mirra et al., 2021). It is expressed predominantly within the cerebellum, hippocampus, cerebral cortex, and retina, playing a crucial role in the function of the latter (García-Arroyo et al., 2021, 2022). Mutations within CERKL cause retinal dystrophies characterized by the progressive degeneration of retinal neurons, ultimately leading to vision loss (Tuson et al., 2004; Aleman et al., 2009; Sen et al., 2020). Two consecutive substitutions, F448L and V449L, within the C-terminal region of the Cerkl protein likely do not significantly impair its function because they are conservative (hydrophobic amino acid residues are replaced by hydrophobic ones). However, their potential role in the development of early neurodegenerative changes in the OXYS rat retina cannot be entirely ruled out

The R276Q substitution within Bfsp1 may accelerate lens degeneration by reducing the mechanical stability of lens intermediate filaments. Dysfunction of BFSP1 is associated with the development of senile cataracts in humans (Song et al., 2009). A charge-depleting amino acid substitution within BFSP1 (D348N) has been reported to cause congenital cataracts in humans (Wang et al., 2023). Bfsp1-knockout mice develop progressive cataracts accompanied by a loss of lens optical properties (Song et al., 2009). Therefore, it can be hypothesized that the R276Q substitution, resulting in the loss of the positive amino acid charge, disrupts the assembly of intermediate filaments and may contribute to the early development of cataracts in OXYS rats – a trait linked to the inheritance of premature aging syndrome, for which these animals were originally selected (Kolosova et al., 2014).

**Muscle pathologies: sarcopenia and cardiomyopathy**

The R815W substitution within the SR1 domain of Akap6 may also contribute to the development of muscle pathologies. AKAP6 has been shown to play a key role in skeletal muscle regeneration by interacting with myogenin, and its inhibition leads to impaired muscle fiber formation and decreased motor activity in mice (Lee et al., 2015). Furthermore, via its spectrin repeats, AKAP6 functions as an adapter protein in cardiomyocytes, linking the nuclear membrane to the microtubuleorganizing center, which is critical for normal cardiac function (Vergarajauregui et al., 2020).

The R3671W substitution within Cmya5 is located within the FN3-1 domain. It disrupts intradomain interactions with other amino acid residues, potentially weakening interactions with titin and destabilizing sarcomeres in both skeletal and cardiac muscle tissues (Sarparanta, 2008; Sarparanta et al., 2010). Disruption of the structural integrity of myofibrils may contribute to the development of sarcopenia and hypertrophic cardiomyopathy, both of which are characteristic of OXYS rats (Vays et al., 2014; Muraleva et al., 2017; Lu et al., 2022).

**Lipid metabolism disorders**

Lipid metabolism disorders are known to significantly contribute to the pathogenesis of AD (Di Paolo, Kim, 2011). The conformation of ApoB-100 in the heparin-binding domain determines the efficiency of the interaction between LDL particles and extracellular matrix proteoglycans in the vascular subendothelial layer. Low-density lipoprotein particles undergo modifications, such as aggregation and oxidation, which trigger inflammatory cascades and enhance atherogenesis (Öörni, Lorey, 2025). The L3413F substitution within this domain of ApoB in OXYS rats could alter this function, potentially contributing to dyslipidemia, systemic oxidative stress, and AD progression. However, based on the analysis of amino acid conservation and protein structure prediction, we assess its effect as minor. It is known that during the period of active manifestation of AD symptoms, dyslipidemia and oxidative stress are not detected in OXYS rats, and blood cholesterol levels are even reduced (Tikhonova et al., 2014). Nonetheless, vascular dysfunction has been shown to significantly contribute to the development of AD (Stefanova et al., 2018), AMD (Zhdankina et al., 2023), and hypertrophic cardiomyopathy (Muraleva et al., 2017) in OXYS rats. The identified V695L substitution within Abca1 is likely functionally insignificant, as it is located within the hydrophobic transmembrane domain and does not disrupt its structure. Nevertheless, it should be noted that ABCA1 plays a key role not only in brain lipid metabolism but also in beta-amyloid clearance, and its genetic variations significantly contribute to AD development (Holstege et al., 2022).

## Conclusion

Whole-genome sequencing with high coverage (>25×) performed on three OXYS rats and three Wistar/Icgn control rats detected genetic variants fixed in the homozygous state in the OXYS strain. Based on comparative analysis, we identified unique variants, absent from other rat strains, which may contribute to the premature aging phenotype and the early onset of age-associated pathologies in OXYS rats. Regarding neurodegeneration, the potentially pathogenic variants include the large deletion within the Fat1 enhancer, leading to the dysregulation of gene expression during neurogenesis; the missense substitution within Cic, potentially disrupting the balance of neural progenitor proliferation and differentiation; variants within Akap6 and Nqo2 associated with cognitive impairment; and the deletion within the Gsta6 promoter that may weaken the antioxidant defense in the brain. Furthermore, the early development of retinopathy may be driven by the deletion within the Abcf1 promoter region, which destabilizes retinal homeostasis, alongside the missense substitutions within Cerkl, potentially reducing the protection of photoreceptors and RPE cells against oxidative stress. The missense substitution within Bfsp1 may impair intermediate filament assembly, leading to cataract development. Finally, the pathogenesis of cardiomyopathy and sarcopenia is likely linked to the missense variant within Cmya5, which impairs myofibril stabilization, and the missense variant within Akap6, which may affect skeletal muscle regeneration and the structural organization of cardiomyocytes. The identified candidate alleles provide a firm basis for future functional analyses and further study of the molecular mechanisms underlying premature aging in OXYS rats.

## Conflict of interest

The authors declare no conflict of interest.
